# ﻿A new genus of Orgyiini (Lepidoptera, Erebidae, Lymantriinae) from China, with description of a new species

**DOI:** 10.3897/zookeys.1243.143534

**Published:** 2025-06-25

**Authors:** Chu-Hang Qiao, Yong-Qiang Xu, Hou-Shuai Wang

**Affiliations:** 1 Department of Entomology, College of Plant Protection, South China Agricultural University, Guangzhou 510642, China South China Agricultural University Guangzhou China; 2 Institute of Plateau Biology of Xizang Autonomous Region, Lhasa 850001, China Institute of Plateau Biology of Xizang Autonomous Region Lhasa China; 3 Motuo Biodiversity Observation and Research Station of Xizang Autonomous Region, Linzhi 860700, China Motuo Biodiversity Observation and Research Station of Xizang Autonomous Region Linzhi China

**Keywords:** *Cyclomaculamedogensis*, DNA barcode, genitalia, identification key, new combination, type species

## Abstract

A new genus, *Cyclomacula* Qiao & Wang, **gen. nov.**, is established to accommodate a new species and three new combinations from China: *C.medogensis* Qiao & Wang, **sp. nov.**, *C.glaucinoptera* (Collenette, 1934), **comb. nov.**, *C.dudgeoni* (Swinhoe, 1907), **comb. nov.** and *C.flavimacula* (Moore, 1865), **comb. nov.** Images of adults and genitalia of all currently recognized *Cyclomacula* species are provided, with illustrations of wing venation of the type species in the new genus. A key to species of the genus is presented, along with DNA barcode data.

## ﻿Introduction

The tribe Orgyiini, belonging to the subfamily Lymantriinae of the family Erebidae, is primarily distributed in the Old World and Nearctic Realm (Wang et al. 2015). It is characterized by dorsal brushes on the first four larval abdominal segments and an accessory cell (also called an areole) in the forewings (Ferguson 1978; Holloway 1999). The tribe was originally proposed by Wallengren (1861) and was later revised by Ferguson (1978), who assigned four genera to Orgyiini based on Nearctic taxa. Subsequently, Holloway (1999) further revised the tribe and recorded nine genera from Borneo. Wang et al. (2015) reconstructed the molecular phylogeny of sixteen Orgyiini genera using eight gene regions.

In the present study, we propose a new genus to accommodate a new species and three species previously classified under the complex genus *Dasychira* Hübner, 1809. We provide illustrations of adults and genitalia of the currently recognized species of the new genus, as well as wing venation of the type species. We also provide a key and DNA barcode data for the species of this genus.

## ﻿Material and methods

### ﻿Examined specimens

The type specimens of *Cyclomaculaglaucinoptera* (Collenette, 1934), comb. nov. and *C.flavimacula* (Moore, 1865), comb. nov. are from the Natural History Museum in London, UK. The other specimens were collected by light trap and were deposited at the Insect Collection, Department of Entomology, College of Plant Protection, South China Agricultural University, Guangzhou, China. Photos of male adults were taken with a NIKON CoolPix S7000 digital camera. The male genitalia were examined after the abdomen was removed and boiled in 10% NaOH solution for 3–5 minutes. The genital slides were photographed with a Carl Zeiss Stemi 2000-CS stereoscope. All photos were processed with Adobe Photoshop CC2023 and SAI ver. 2 software. The terminology of adults and genitalia follows Ferguson (1978), Holloway (1999) and Chao (2003).

### ﻿Molecular data analyses

Total genomic DNA was extracted from two or three legs of adults using a TIANamp Genomic DNA Kit (Tiangen Biotech Co. Ltd, Beijing, China). A 658 bp fragment of the mitochondrial gene cytochrome *c* oxidase I (COI), namely DNA barcode, was amplified from the new species and *Cyclomaculaglaucinoptera* comb. nov., with the universal primers LCO1490 and HCO2198 (Folmer et al. 1994). DNA extraction and PCR amplification followed Wang et al. (2014). The DNA barcode sequences of *C.dudgeoni* comb. nov. and *C.flavimacula* comb. nov. were sourced from Wang’s research (2015). Other *Cyclomacula* and species of other genera were downloaded from the NCBI database. The detailed sampling data in this study are listed in Table 1. The pairwise genetic distances of the *COI* gene in these species were calculated under the Kimura 2-Parameter (K2P) model (Kimura 1980) in the MEGA11 software (Tamura et al. 2021).

**Table 1.** Sampling data used in the molecular analysis.

Taxa	Locality	Date	GenBank	Source
*Cyclomaculaflavimacula* **comb. nov.**	China	-	KP081879.1	NCBI, Wang et al. (2015)
*Cyclomaculadudgeoni* **comb. nov.**	China	-	KP081880.1	NCBI, Wang et al. (2015)
*Cyclomaculaglaucinoptera* **comb. nov.**	China	2022-10-02	PQ530438.1	NCBI, newly sequenced in this study
*Cyclomaculaglaucinoptera* **comb. nov.**	China	2023-11-01	PQ530437.1	NCBI, newly sequenced in this study
*Dasychiratephra*	USA	-	KP081877.1	NCBI, Wang et al. (2015)
*Olenemendosa*	India	-	OQ825993.1	NCBI, Phom and Somasundaram (2024)
*Telochurusrecens*	Canada	2010-01-01	JF853960.1	NCBI

“-” represents missing data.

## ﻿Results

The genetic distances of *COI* among the species of *Cyclomacula* gen. nov. and the type species of three closely related genera are listed in Table 2. The pairwise distances between species of the new genus and the type species of its closely related genera range from a minimum of 0.110 between *Cyclomaculadudgeoni* comb. nov. and *Dasychiratephra* Hübner, 1809 to a maximum of 0.163 between *Cyclomaculaglaucinoptera* comb. nov. and *Olenemendosa* Hübner, 1823. The mean genetic distance among *Cyclomacula* species is 10.7%. The minimal interspecific genetic distance within the new genus is between species *C.medogensis* sp. nov. and *C.glaucinoptera* comb. nov. with a value of 8.2%.

**Table 2.** Pairwise genetic distances of COI sequences for all taxa used in this study.

Species code	Species name	1	2	3	4	5	6
**1**	*Cyclomaculaalbilium* **sp. nov.**						
**2**	*Cyclomaculaglaucinoptera* **comb. nov.**	0.082					
**3**	*Cyclomaculaflavimacula* **comb. nov.**	0.105	0.112				
**4**	*Cyclomaculadudgeoni* **comb. nov.**	0.108	0.115	0.122			
*5*	*Olenemendosa*	0.146	0.163	0.158	0.171		
*6*	*Dasychiratephra*	0.116	0.126	0.140	0.110	0.176	
*7*	*Telochurusrecens*	0.113	0.122	0.126	0.133	0.159	0.101

### ﻿Key to species of *Cyclomacula* gen. nov. in China based on the male

**Table:** 

1	Forewings without a yellow-white crescent spot near anal angle	**2**
–	Forewings with a yellow-white crescent spot near anal angle	***C.flavimacula* comb. nov.**
2	Valvae with a triangle sclerotized saccular process	**3**
–	Valvae with an indistinct saccular process	***C.medogensis* sp. nov.**
3	Valvae wide, about half the base, narrowed at about the distal half	***C.dudgeoni* comb. nov.**
–	Valvae wide, about 2/3 the base, narrowed at about the distal 1/3	***C.glaucinoptera* comb. nov.**

#### ﻿ Cyclomacula gen. nov.

Taxon classificationAnimaliaLepidopteraErebidae

BD71B1B5-C130-58AD-9327-2F44A0199F86

https://zoobank.org/89844E6C-A08B-456A-AFA9-9217456C7364

##### Type species.

*Dasychiraglaucinoptera* Collenette, 1934, by present designation.

##### Diagnosis.

The new genus usually possesses a nearly ring-like basal spot on the male forewings. It can be easily distinguished from the type species of *Dasychira* and *Olene* Hübner, 1823 (*D.tephra* and *O.mendosa*) by valvae without branching into two arms in the male genitalia. The new genus is also similar to *Telochurus* Maes, 1984, but can be separated from the latter by the aedeagus without a cornutus.

##### Description.

**Figure 1.**
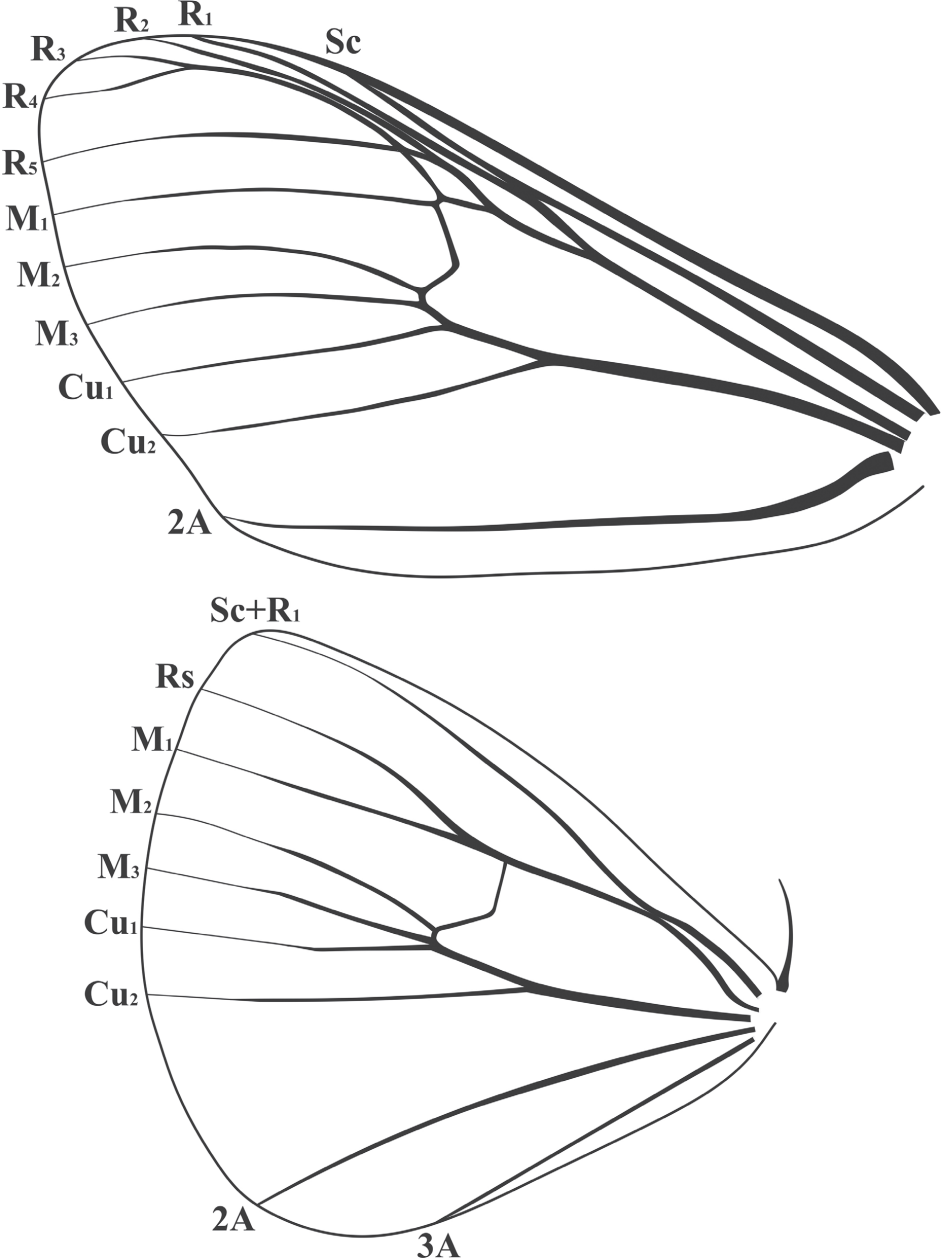
Wing venation of *Cyclomaculaglaucinoptera* comb. nov., male.

Labial palpi short and brown. Antennae bipectinate, stronger in male compared to female. Forewings usually with a ring-like basal spot in male, and an irregular, sometimes wavy oblique postmedial fascia in female; wing venation (Fig. 1) with an accessory cell; R_1_ branching from distal 1/3 of dorsal margin of discal cell, R_2_, R_3+4_ and R_5_ arising from accessory cell, respectively; M_1_ originated from upper angle of discal cell, M_2_ and M_3_ originated from lower angle of discal cell, respectively; Cu_1_ parallel to Cu_2_. Hindwings without markings in male, but an indistinct postmedian fascia often present in female; Rs short stalked with M_1_; M_2_ present, arising from lower angle of discal cell; Cu_1_ parallel to Cu_2_.

***Male genitalia*.** Uncus short, digitate. Gnathus bifid, each fork crescent-shaped. Valvae board basally, narrowed distally. Saccus small. Aedeagus simple.

***Female genitalia*.** Anal papillae broad. Apophyses well developed. Ductus poorly sclerotized at base. Bursa large, signum sclerotized medially or absent.

##### Etymology.

The new genus is named after the Latin words “*cyclus*” and “*macula*”, which refer to the ring-like basal spot on the forewings.

##### Distribution.

China (Jiangsu, Zhejiang, Hubei, Hunan, Fujian, Guangdong, Guangxi, Hainan, Shaanxi, Gansu, Sichuan, Yunnan, Xizang); India, Nepal, Malaysia, Indonesia.

#### ﻿ Cyclomacula medogensis

Taxon classificationAnimaliaLepidopteraErebidae

Qiao & Wang sp. nov.

2C585992-FDEA-5484-B41F-41E276649FE3

https://zoobank.org/C6ACCD0C-617C-4938-A0F6-5F8A5E0A93C1

Figs 2A, B , 4A

##### Type materials.

***Holotype***: China • ♂; Xizang Autonomous Region, Linzhi City, Medog County; alt. 2400 m; 1 Nov. 2023; Chuhang Qiao & Ziqi Yuan & Liang Guo leg. ***Paratype***: • 1♂; same collection data as for preceding.

##### Diagnosis.

The new species is similar to *C.glaucinoptera* (Collenette, 1934), comb. nov. in male, but can be distinguished from the latter by the hindwings light brown, uncus slightly pointed, valvae with a weak and indistinct saccular process. It also resembles *C.dudgeoni* comb. nov. in male, but the latter has dark brown wings and longer valvae. The new species differs from *C.flavimacula* comb. nov. by forewings without a yellow-white crescent spot near anal angle, and gnathus bifurcating into two slenderer forks.

##### Description.

**Figure 2.**
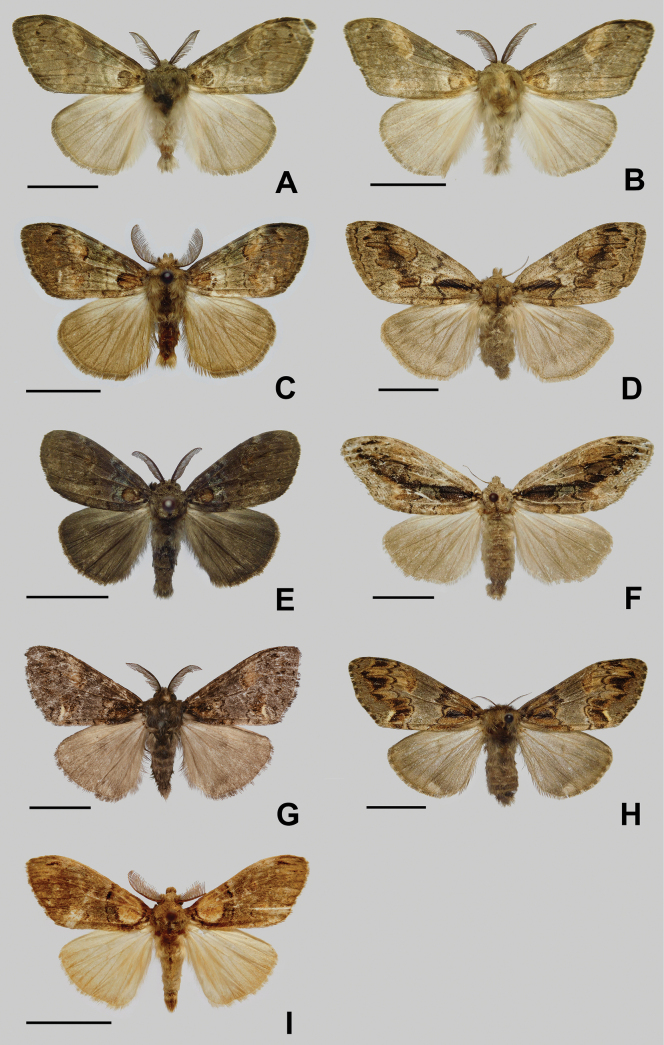
Adults of *Cyclomacula* species (**A–H**) and *Olene* species (**I**). **A.***Cyclomaculamedogensis* sp. nov. holotype, male; **B.***C.medogensis* sp. nov. paratype, male; **C.***C.glaucinoptera* comb. nov., male; **D.***C.glaucinoptera* comb. nov., female; **E.***C.dudgeoni* comb. nov., male; **F.***C.dudgeoni* comb. nov., female; **G.***C.flavimacula* comb. nov., male; **H.***C.flavimacula* comb. nov., female; **I.***Olenemendosa*, male. Scale bars: 10 mm.

***Male adult*** (Fig. 2A, B). Forewing length: 19–20 mm. Antennae bipectinate, dark brown. Vertex and frons with greyish hair. Labial palpi yellowish-brown. Thorax dark brown or yellowish-brown. Abdomen light brown. Forewings ground color greyish-brown, with two dark brown or yellowish-brown basal spots in which the ventral one is nearly ring-like; a large, faint yellow crescent spot present along the outer margin of discal cell slanting to costa; antemedian line blurred, dark brown; postmedian fascia wavy; and cilia of outer margin dark brown. Hindwings light brown, without markings; cilia of outer margin dark brown.

***Male genitalia*** (Fig. 4A). Uncus short, digitate. Gnathus well developed, bifid, each fork crescent-shaped. Valvae broad at basal half, narrowed at distal half. Juxta well sclerotized at dorsal margin. Saccus small. Aedeagus simple, slightly curved distally.

##### Etymology.

The species is named after its type locality: Medog County.

##### Distribution.

China (Xizang Autonomous Region).

#### ﻿ Cyclomacula glaucinoptera

Taxon classificationAnimaliaLepidopteraErebidae

(Collenette, 1934) comb. nov.

D5ECA9D8-70F2-5412-A14C-200C35E5E49D

**Figure 3.**
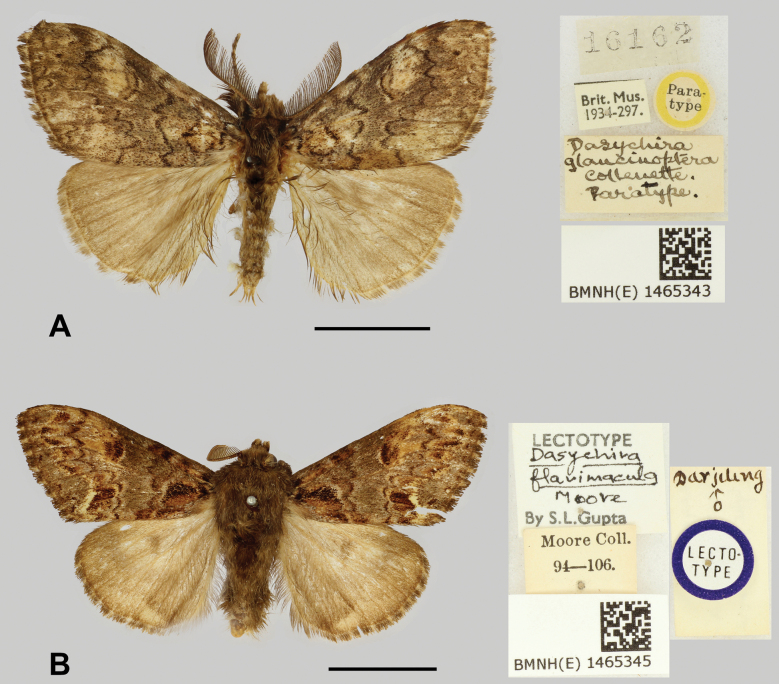
Type materials of *Cyclomacula*. **A.***C.glaucinoptera* comb. nov. (paratype, male, copyright from Natural History Museum in London); **B.***C.flavimacula* comb. nov. (lectotype, male, copyright from the Natural History Museum in London). Scale bars: 10 mm.

**Figure 4.**
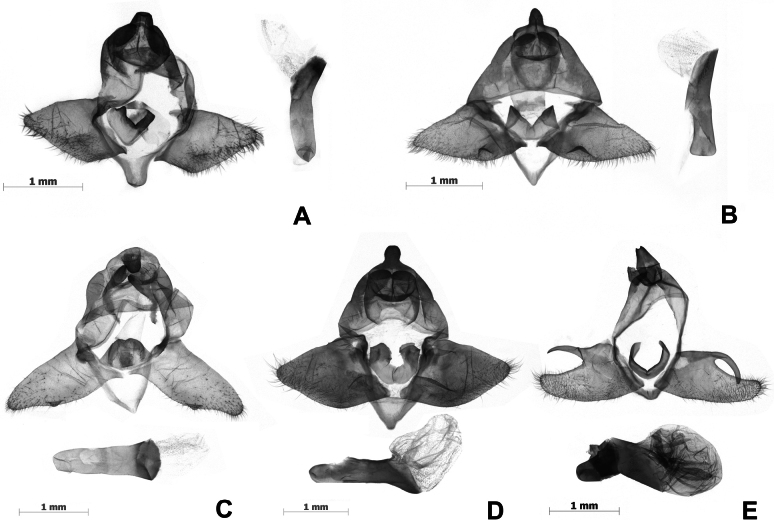
Male genitalia of *Cyclomacula* species (**A–D**) and *Olene* species (**E**). **A.***Cyclomaculamedogensis* sp. nov.; **B.***C.glaucinoptera* comb. nov.; **C.***C.dudgeoni* comb. nov.; **D.***C.flavimacula* comb. nov.; **E.***Olenemendosa*. Scale bars: 1 mm.

**Figure 5.**
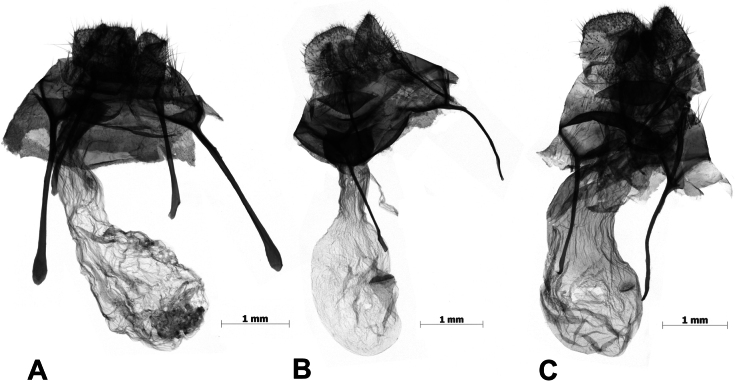
Female genitalia of *Cyclomacula*. **A.***C.glaucinoptera* comb. nov.; **B.***C.dudgeoni* comb. nov.; **C.***C.flavimacula* comb. nov. Scale bars: 1 mm.

Figs 2C, D , 3A , 4B , 5A

Dasychira glaucinoptera Collenette, 1934: 117; Chao 2003: 94.

##### Material examined.

China • 1♂, ***paratype*** of *C.glaucinoptera*; Zhejiang Province, Hangzhou City, West Tianmu Mountian; alt. 1600 m; 25 Jul. and Oct. 1931; H. Höne leg (Fig. 3A). Hubei Province • 1♂1♀; Yichang City; 20 Oct. 2012; Min Wang leg. Shaanxi Province • 2♂; Baoji City, Feng County; alt. 1500 m; 22 Sep. 2022; Liping Zhou leg.; • 1♂2♀; same collection data as for preceding; 2 Oct. 2023. Sichuan Province • 1♂; Yaan City, Yingjing County, Longcanggou National Forest Park; 25 Jul. 2015; Min Wang leg.

##### Redescription.

***Male adult*** (Fig. 2C). Forewing length: 19–22 mm. Antennae bipectinate, dark brown. Vertex and frons with light greyish-brown hair. Labial palpi light greyish-brown. Thorax and abdomen greyish-brown. Forewings ground color greyish-brown, with two reddish-brown basal spots in which the ventral one is nearly ring-like; a black line present along the outer margin of discal cell; antemedian and postmedian line dark brown, wavy; cilia of outer margin dark brown. Hindwings brown, without markings; cilia of outer margin brown.

***Female adult*** (Fig. 2D). Forewing length: 25–27 mm. Antennae bipectinate, greyish-brown. Vertex and frons with greyish hair. Labial palpi greyish-brown. Thorax greyish. Abdomen light greyish-brown. Forewings greyish-brown, with a basal stripy black spot; antemedian line black; postmedian fascia broad, irregular, ending at vein 2A; outer margin with a black line, cilia dark brown. Hindwings light greyish-brown, with an indistinct submarginal dark brown band.

***Male genitalia*** (Fig. 4B). Uncus short, digitate. Gnathus well developed, bifid, each fork crescent-shaped. Valvae broad at basal half, narrowed at distal half, with a triangle sclerotized saccular process about the middle of the ventral margin. Saccus small, triangular. Aedeagus relatively straight, with tiny spines terminally.

***Female genitalia*** (Fig. 5A). Anal papillae broad. Apophyses anterioris slightly longer than apophyses posterioris. Ductus poorly sclerotized at base. Bursa broad and thin without signum.

##### Distribution.

China (Zhejing, Hubei, Fujian, Sichuan, Yunnan, Xizang, Shaanxi, Gansu).

#### ﻿ Cyclomacula dudgeoni

Taxon classificationAnimaliaLepidopteraErebidae

(Swinhoe, 1907) comb. nov.

1FA732ED-C7A1-5313-BDA8-E37297D29538

Figs 2E, F , 4C , 5B

Dasychira dudgeoni Swinhoe, 1907: 203; Chao 2003: 90.
Pseudodura dasychiroides Strand, 1914: 333.
Orgyia dudgeoni Swinhoe 1923: 404.
Olene dudgeoni Holloway 1999: 33.

##### Material examined.

China • 1♂; Fujian Province, Wuyishan City, Wuyishan National Park; alt. 1200 m; 17 May 2021; Min Wang & Chuyang Huang leg. Guangdong Province • 4♂1♀; Huizhou City, Longmen County, Nankun Mountain; 10 Sep. 2024; Chuhang Qiao & Ziqi Yuan leg. Guangdong Province • 2♂; Shaoguan City, Ruyuan County, Nanling National Nature Reserve; 31 May 2017; Min Wang leg. Hainan Province • 1♂1♀; Jianfengling National Nature Reserve; 9 Jul. 2018; Shifang Mo & Houshuai Wang & Zhipeng Miao leg. Zhejiang Province, • 1♂; Hangzhou City, Linan County, Tianmu Mountain; alt. 1080 m; 14 Jun. 2015; Min Wang leg.

##### Redescription.

***Male adult*** (Fig. 2E). Forewing length: 14–19 mm. Antennae bipectinate, dark brown. Vertex and frons with greyish-brown hair. Labial palpi dark brown. Thorax dark brown. Abdomen greyish-brown. Forewings dark brown, with a ring-like reddish-brown basal spot; antemedian fascia dark blue, postmedian fascia inconspicuous. Hindwings greyish-brown.

***Female adult*** (Fig. 2F). Forewing length: 20–27 mm. Antennae bipectinate, greyish-brown. Vertex, frons and labial palpi greyish-brown. Thorax greyish-brown. Abdomen dark khaki. Forewings ground color greyish-brown, with a broad black patch at base, antemedian and postmedian lines black, postmedian fascia broad, black. Hindwings dark khaki, without markings.

***Male genitalia*** (Fig. 4C). Uncus short, digitate. Gnathus bifid, each fork crescent-shaped. Valvae broad about the basal 2/3, narrowed about the distal 1/3, with a small triangular sclerotized saccular process near the distal 1/3 of the ventral margin. Saccus small and triangular. Aedeagus straight, inflated terminally.

***Female genitalia*** (Fig. 5B). Anal papillae broad. Apophyses anterioris slightly longer than apophyses posterioris. Ductus short. Bursa large, signum with a transverse stripe-shaped sclerotization in middle.

##### Distribution.

China (Jiangsu, Zhejing, Hubei, Hunan, Fujian, Guangdong, Guangxi, Hainan, Yunnan); India, Malaysia, Indonesia.

#### ﻿ Cyclomacula flavimacula

Taxon classificationAnimaliaLepidopteraErebidae

(Moore, 1865) comb. nov.

32FD35EA-727E-5BFE-8F88-5E4D805EBE9C

Figs 2G, H , 3B , 4D , 5C

Dasychira flavimacula Moore, 1865: 803–804: Chao 2003: 92.
Orgyia flavimacula Swinhoe, 1923: 298.

##### Material examined.

China • 1♂; Shaanxi Province, Baoji City, Taibai County; alt. 1407 m; 25 Sep. 2019; Min Wang leg. Xizang Autonomous Region • 1♂1♀; Cuona City, Lemenba County, alt. 2400 m, 23 May 2018, Houshuai Wang leg. Yunnan Province • 1♂; Nujiang Lisu Autonomous Prefecture, Gongshan Dulong and Nu Autonomous County, Pukawang village; alt. 1409 m; 4 May 2024; Shaoji Hu leg. Yunnan Province • 1♂1♀; Lijiang City, Yulong Naxi Autonomous County, Yulong Snow Mountain; alt. 3500 m; 18 Jun. 2024; Chuhang Qiao & Ziqi Yuan leg. INDIA • 1♂; ***lectotype*** of *C.flavimacula*, Darjeeling (Fig. 3B).

##### Redescription.

***Male adult*** (Fig. 2G). Forewing length: 19–21 mm. Antennae bipectinate, dark brown. Vertex and frons with greyish-brown hair. Labial palpi reddish-brown. Thorax and abdomen dark brown. Forewings background color greyish-brown with an indistinct basal patch; antemedian and postmedian lines dark brown, wavy; a yellow crescent spot present near anal angle; cilia of outer margin dark brown. Hindwings brown, without markings; cilia of outer margin dark brown.

***Female adult*** (Fig. 2H). Forewing length: 26–27 mm. Antennae bipectinate, greyish-brown. Frons, vertex and labial palpi greyish-brown. Thorax and abdomen greyish-brown. Forewings background color greyish-brown with a basal dark brown patch; postmedian fascia broad, waved; a yellow-brown patch present along the outer margin of discal cell, and a yellow crescent spot present near anal angle; cilia dark brown mixed with dark yellow. Hindwings greyish-brown, without markings; cilia of outer margin dark brown mixed with dark yellow.

***Male genitalia*** (Fig. 4D). Uncus short, digitate. Gnathus bifid and broad, each fork crescent-shaped. Valvae broad at basal half, tapered at distal half. Juxta U-shaped. Saccus small and triangular, pointed at the terminal. Aedeagus straight, without cornutus.

***Female genitalia*** (Fig. 5C). Anal papillae broad. Apophyses anterioris slightly longer than apophyses posterioris. Ductus short with a short-sclerotized base. Bursa broad, with a small transversely sclerotized signum.

##### Distribution.

China (Sichuan, Yunnan, Xizang, Shaanxi); India, Nepal.

## ﻿Discussion

Three of the four recognized species in *Cyclomacula* gen. nov. were previously classified under the very complex genus *Dasychira*. However, the valvae of the type species of *Dasychira*, *D.tephra*, are divided into two arms (Ferguson 1978: fig. 7), which are distinctly different from those of *Cyclomacula* species. Ferguson (1978) confirmed *D.tephra* as a North American species, and asserted that this species is not congeneric with those *Dasychira* species in the Old World; he thus restricted *Dasychira* to a group of species from North America. This taxonomic treatment was subsequently accepted by Holloway (1999) and Wang et al. (2015). We hereby establish a new genus to accommodate the three species in the present study. Sexual dimorphism is observed within the new genus.

*Cyclomaculadudgeoni* comb. nov. was transferred to *Olene* by Holloway (1999) primarily based on wing facies, but he noted a significant difference between this species and *O.mendosa* (the type species of *Olene*; Figs 2I, 4E) in male genitalia. Wang et al. (2015) further confirmed that this taxonomic arrangement was problematic based on their molecular phylogeny of Lymantriinae. The male genitalia of *Cyclomacula* species are similar to those of the type species of *Telochurus* Maes, *T.recens* Hübner, 1819, but the latter possesses a spined, plate-like cornutus (Maes 1984: plate 4, fig. A).

The interspecific genetic distance of the *COI* gene in Lepidoptera is generally greater than 3% (Hebert et al. 2003). Our results show that the minimum genetic distance of *COI* between the new species and all the other *Cyclomacula* species is 8.2%, further supporting the validity of the new species.

## Supplementary Material

XML Treatment for Cyclomacula

XML Treatment for Cyclomacula medogensis

XML Treatment for Cyclomacula glaucinoptera

XML Treatment for Cyclomacula dudgeoni

XML Treatment for Cyclomacula flavimacula

## ﻿Additional information

### Author ORCIDs

Chu-Hang Qiao https://orcid.org/0009-0003-3852-0933

Yong-Qiang Xu https://orcid.org/0000-0002-1889-9531

Hou-Shuai Wang https://orcid.org/0000-0002-0796-9768

### Data availability

All of the data that support the findings of this study are available in the main text.
